# Ultrasmall cerium oxide nanoparticles as highly sensitive X-ray contrast agents and their antioxidant effect†

**DOI:** 10.1039/d3ra08372a

**Published:** 2024-01-23

**Authors:** Abdullah Khamis Ali Al Saidi, Adibehalsadat Ghazanfari, Ahrum Baek, Tirusew Tegafaw, Mohammad Yaseen Ahmad, Dejun Zhao, Ying Liu, Ji-ung Yang, Ji Ae Park, Byeong Woo Yang, Kwon Seok Chae, Sung-Wook Nam, Yongmin Chang, Gang Ho Lee

**Affiliations:** a Department of Chemistry, College of Natural Sciences, Kyungpook National University Taegu 41566 South Korea ghlee@mail.knu.ac.kr; b Institute of Biomedical Engineering, School of Medicine, Kyungpook National University Taegu 41944 South Korea; c Division of Applied RI, Korea Institute of Radiological & Medical Sciences Seoul 01812 South Korea; d Theranocure Medlifescience Bldg. 1, Chilgok, Bukgu Taegu 41405 South Korea; e Department of Biology Education, Teachers' College, Kyungpook National University Taegu 41566 South Korea; f Department of Molecular Medicine, School of Medicine, Kyungpook National University Taegu 41944 South Korea ychang@knu.ac.kr

## Abstract

Owing to their theranostic properties, cerium oxide (CeO_2_) nanoparticles have attracted considerable attention for their key applications in nanomedicine. In this study, ultrasmall CeO_2_ nanoparticles (particle diameter = 1–3 nm) as X-ray contrast agents with an antioxidant effect were investigated for the first time. The nanoparticles were coated with hydrophilic and biocompatible poly(acrylic acid) (PAA) and poly(acrylic acid-*co*-maleic acid) (PAAMA) to ensure satisfactory colloidal stability in aqueous media and low cellular toxicity. The synthesized nanoparticles were characterized using high-resolution transmission electron microscopy, X-ray diffraction, Fourier transform-infrared spectroscopy, thermogravimetric analysis, dynamic light scattering, cell viability assay, photoluminescence spectroscopy, and X-ray computed tomography (CT). Their potential as X-ray contrast agents was demonstrated by measuring phantom images and *in vivo* CT images in mice injected intravenously and intraperitoneally. The X-ray attenuation of these nanoparticles was greater than that of the commercial X-ray contrast agent Ultravist and those of larger CeO_2_ nanoparticles reported previously. In addition, they exhibited an antioxidant effect for the removal of hydrogen peroxide. The results confirmed that the PAA- and PAAMA-coated ultrasmall CeO_2_ nanoparticles demonstrate potential as highly sensitive radioprotective or theranostic X-ray contrast agents.

## Abstract 2

Ultrasmall CeO_2_ nanoparticles as highly sensitive radioprotective or theranostic X-ray contrast agents.

## Graphical abstract

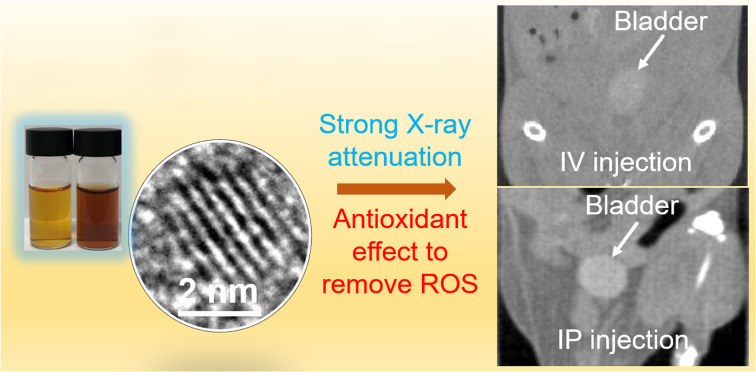

## Introduction

Owing to their excellent physicochemical properties, metal-based nanoparticles have attracted considerable interest in various applications; thus, these nanoparticles provide enticing opportunities to overcome the limitations of existing technologies or to make breakthroughs in a new field.^1–4^ Metal-based nanoparticle contrast agents in X-ray computed tomography (CT) are more sensitive than commercial molecular iodine contrast agents.^5–11^ Therefore, they can provide enhanced diagnosis at reduced doses.

As one of the reliable and prevalent imaging modalities owing to its innate ability to provide high-resolution as well as whole-body scan,^12,13^ CT is based on high-energy ionizing X-ray radiation *via* which free radicals and reactive oxygen species (ROS) can be generated during an X-ray scan.^14–17^ The natural radiation dose is 2–3 mSv per year.^18^ Each medical CT scan covers 0.001–16 mSv, depending on the scanning objects of the body; hence, multiple CT scans are harmful to the body.^19^ Contrast agents can reduce the X-ray radiation dose without deteriorating the image quality *via* contrast enhancement.^12,13^ They also facilitate the identification and diagnosis of certain conditions and diseases of the body.^12,13^ Currently, the iodine contrast agents approved by the United States Food & Drug Administration^6,20^ exhibit limitations, such as low sensitivity, necessitating high injection doses that could cause side effects,^21^ and low contrast for soft tissues. In addition, they undergo rapid renal excretion because of their low molecular masses, allowing only brief imaging times. However, heavy metal-based nanoparticles can overcome these limitations because of their higher X-ray attenuation,^22^ lower osmolality and viscosity,^6,23^ and longer blood vessel circulation times^24^ than those of molecular iodine contrast agents, leading to higher contrast images, lower injection doses, and longer imaging times. Therefore, developing alternative contrast agents derived from heavy metal-based nanoparticles is imperative.

In particular, cerium oxide (CeO_2_) nanoparticles exhibit an additional unique property of reducing the ionizing risks of X-rays *via* their antioxidant effect based on feasible oxidation state interconversion between Ce^3+^ and Ce^4+^.^14–16,25,26^ CeO_2_ nanoparticles can scavenge free radicals and ROS produced during CT scans, thereby protecting against tissue damage.^26–28^ This property of CeO_2_ nanoparticles further renders antibacterial and antineurodegenerative therapeutic properties.^29–31^

Thus far, a limited number of Ce-containing nanoparticles have been reported as radioprotective^15^ or theranostic^32–36^ X-ray contrast agents. Based on the high X-ray attenuation of CeO_2_ nanoparticles^22^ and their exceptional catalytic properties, rendering them highly effective in removing excess ROS from radiation-induced damage,^26–28^ Garcia *et al.* synthesized 5 nm albumin-stabilized CeO_2_ nanoparticles and used them for the *in vivo* imaging of normal and tumor-model mice.^15^ Chaurand *et al.* successfully located CeO_2_ nanomaterials [particle diameter (*d*) = ∼31 nm] in mouse lung tissue using X-ray imaging.^32^ They reported that the X-ray attenuation was ∼2 times greater than that of the commercial iodine contrast agent Iohexol. Liu *et al.* synthesized CeO_*x*_ nanoparticles embedded in mesoporous silica particles (overall diameter = 119–134 nm) and applied them for the diagnosis and X-ray induced photodynamic therapy of cancer.^33^ They reported that the X-ray attenuation was 3.79 times greater than that of the iodine contrast agent Iohexol. Cao *et al.* synthesized dextran-coated CeO_2_ nanoparticles (*d* = 3 nm) and applied them to CT-guided therapy of inflammatory bowel disease by scavenging ROS and down-regulating proinfammatory cytokines.^34^ Naha *et al.* synthesized dextran-coated CeO_2_ nanoparticles (*d* = 4.8 nm) and applied them to CT diagnosis of gastrointestinal tract and inflammatory bowel disease.^35^ The X-ray attenuation was ∼1.2 times greater than that of the commercial iodine contrast agent Iopamidol. Jia *et al.* synthesized doxorubicin-loaded upconversion core@mesoporous CeO_*x*_ shell nanoplatforms (*d* = ∼48 nm) for tumor diagnosis *via* CT and the synergistic chemophotodynamic therapy of tumor.^36^ Feng *et al.* synthesized citric acid-coated CeO_2_ nanoparticles (*d* = ∼3 nm) as a renoprotective contrast agent and successfully applied them to *in vivo* spectral CT angiography.^37^ Youn *et al.* synthesized CeO_2_ nanoparticles (*d* = 3.5 nm) and nanorods (9.4 × 130 nm), and compared their therapeutic effects. Compared to the nanoparticles, the nanorods demonstrated better effects on reducing cerebral edema.^38^

Herein, ultrasmall CeO_2_ nanoparticles (*d* = 1–3 nm) coated with hydrophilic and biocompatible polymers, namely, poly(acrylic acid) (PAA) and poly(acrylic acid-*co*-maleic acid) (PAAMA), were synthesized using the one-pot polyol method. Their particle diameters were less than those^32–39^ of the previously investigated nanoparticles. Notably, smaller CeO_2_ nanoparticles in particle size can exhibit a higher X-ray attenuation efficiency due to their more effective X-ray attenuation and more powerful antioxidant effect because of their higher amounts of Ce^4+^ on nanoparticle surfaces. Therefore, ultrasmall CeO_2_ nanoparticles synthesized herein can act as highly sensitive radioprotective or theranostic X-ray contrast agents. The polymer-coated ultrasmall CeO_2_ nanoparticles were characterized using various techniques. Cellular cytotoxicity was assessed to confirm their suitability for biomedical applications. The X-ray attenuation properties were characterized by measuring phantom images. The CT images *in vivo* were measured before and after intravenous (IV) and intraperitoneal (IP) injections to confirm the potential of the CeO_2_ nanoparticles as X-ray contrast agents. Finally, their antioxidant effect was evaluated by measuring the removal of hydrogen peroxide (H_2_O_2_) in the oxidation reaction of rhodamine B (Rh B) under H_2_O_2_/365 nm ultraviolet (UV) irradiation with and without the nanoparticles.

## Results and discussion

### Colloidal stability, particle diameter, hydrodynamic diameter, zeta potential, and crystallinity

The PAA- and PAAMA-coated ultrasmall CeO_2_ nanoparticles, exhibiting colloidal stability, were successfully prepared using a simple one-pot polyol method (Fig. S1†), as confirmed by the below-described characterization methods.

**Fig. 1:**
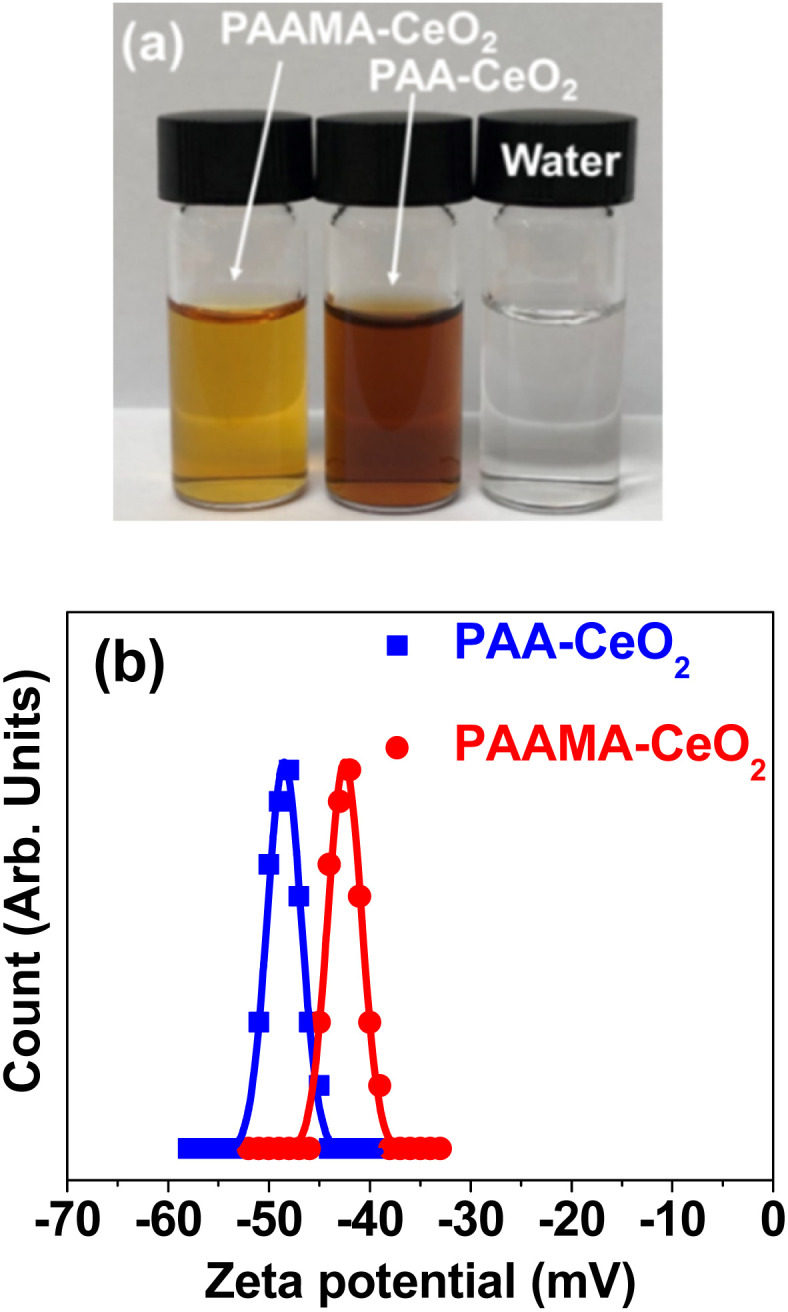
(a) Photographs of PAA- and PAAMA-coated ultrasmall CeO_2_ nanoparticles dispersed in aqueous media and water. (b) Zeta potential (*ζ*) curves and Gaussian function fits to obtain *ζ*_avg_.

Transparent nanoparticles were suspended in aqueous media, which did not undergo precipitation after synthesis (>1.5 years), indicating excellent colloidal stability (Fig. 1a). The high negative average zeta potentials (*ζ*_avg_) of −48.3 and −43.0 mV for the PAA- and PAAMA-coated ultrasmall CeO_2_ nanoparticles in aqueous media, respectively (Fig. 1b and Table 1), confirmed their excellent colloidal stability in aqueous media. The colloidal dispersion was also confirmed by Tyndall effect (Fig. S2†); light scattering was observed only for nanoparticle suspension samples owing to the collision between the nanoparticle colloids and laser light, whereas light scattering was not observed in triple-distilled water.

**Table 1:** Physicochemical properties of PAA- and PAAMA-coated ultrasmall CeO_2_ nanoparticles

Surface-coating polymer	*d*_avg_ (nm)	*a*_avg_ (nm)	*ζ* (mV)	Surface-coating		
				*S* a (wt%)	*σ* b (nm^−2^)	*N*_polymer_ c
PAA	1.8	14.5	−48.3	56	1.2	12
PAAMA	2.0	15.5	−43.0	37	0.3	4

a Average coating amount of polymers per nanoparticle in wt%.

b Grafting density, *i.e.*, average number of polymers coating a nanoparticle unit surface area.

c Average number of polymers coating a nanoparticle.

**Fig. 2:**
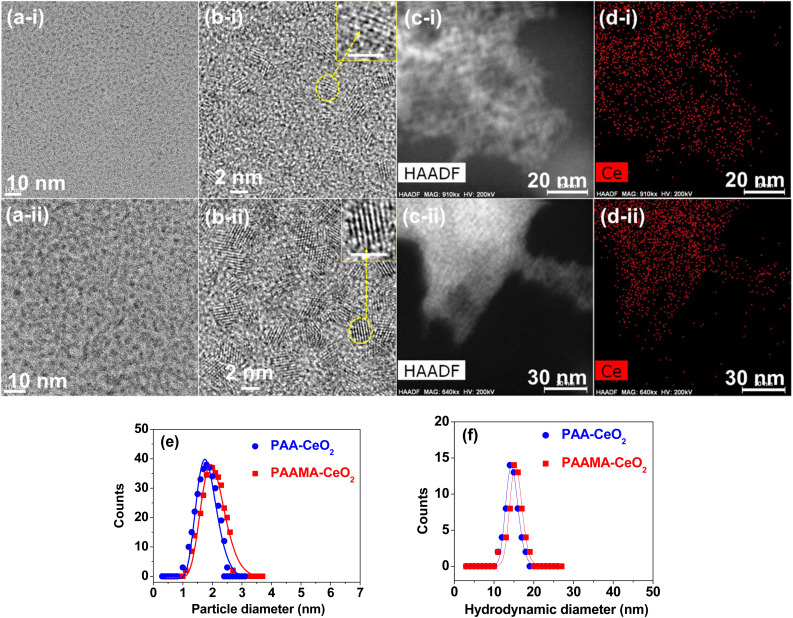
(a(i)), (a(ii)), (b(i)), and (b(ii)) HRTEM images: nanoparticles enclosed within the dotted circles in (b(i)) and (b(ii)) were magnified as indicated by the arrows (scale bar = 2 nm). (c(i)) and (c(ii)) HAADF-STEM images. (d(i)) and (d(ii)) Elemental mapping in the HAADF-STEM mode. (e) Particle diameter distributions and log–normal function fits to obtain *d*_avg_. (f) DLS patterns and log–normal function fits to obtain *a*_avg_. In (a)–(d), (i) indicates PAA-coated ultrasmall CeO_2_ nanoparticles and (ii) indicates PAAMA-coated ultrasmall CeO_2_ nanoparticles.

High-resolution transmission electron microscopy (HRTEM) images of polymer-coated CeO_2_ nanoparticles revealed nearly monodisperse particle diameter distributions (Fig. 2a(i), a(ii), b(i) and b(ii)) in which (i) and (ii) label PAA- and PAAMA-coated ultrasmall CeO_2_ nanoparticles, respectively. Additional HRTEM images are provided in ESI (Fig. S3 and S4†). The nanoparticle dispersions were confirmed by elemental mapping in the high-angle annular dark field-scanning transmission electron microscope (HAADF-STEM) mode (Fig. 2c(i) and (ii)), which revealed the uniform elemental distribution of Ce (Fig. 2d(i) and (ii)) in HAADF-STEM images. X-ray energy dispersive spectroscopy spectra (Fig. S5a and b†) confirmed the presence of Ce in the nanoparticles. The average particle diameters (*d*_avg_) for PAA- and PAAMA-coated ultrasmall CeO_2_ nanoparticles were estimated to be 1.8 and 2.0 nm, respectively, based on the log–normal function fits to the observed particle diameter distributions (Fig. 2e and Table 1). The average hydrodynamic diameter (*a*_avg_) values of the PAA- and PAAMA-coated ultrasmall CeO_2_ nanoparticles were estimated to be 14.5 and 15.5 nm, respectively, based on the log–normal function fits to the observed dynamic light scattering (DLS) patterns (Fig. 2f). The large hydrodynamic diameter of the nanoparticles was attributed to the PAA and PAAMA coatings on the nanoparticle surfaces and accompanying hydration of a large amount of water. Each monomer in PAA comprises one carboxyl group. PAAMA comprises almost equal numbers of acrylic acid (AA) and maleic acid (MA) monomers, and each of the AA and MA monomers comprises one and two carboxyl groups, respectively. These numerous carboxyl groups possibly lead to strong binding between the polymers and nanoparticles *via* electrostatic (*i.e.*, hard acid–base) interaction, consequently supporting their observed excellent colloidal stability in aqueous media.

**Fig. 3:**
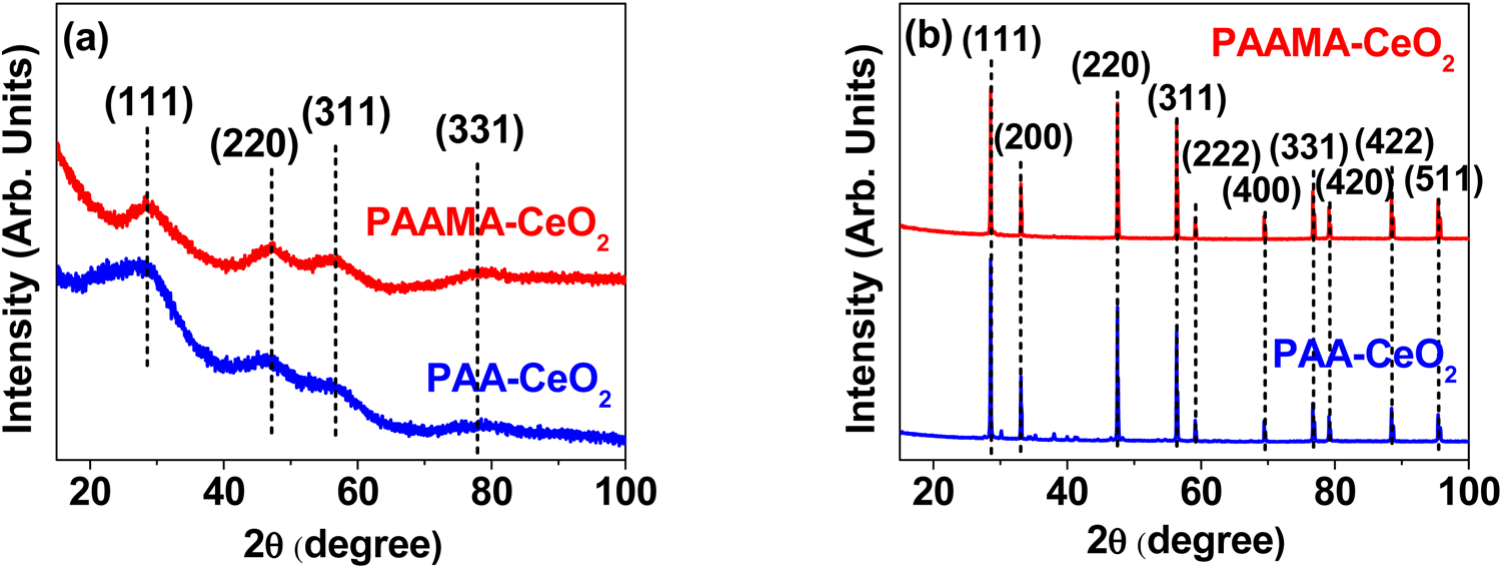
XRD patterns of the powder samples of the PAA- and PAAMA-coated ultrasmall CeO_2_ nanoparticles (a) before and (b) after TGA up to 900 °C under airflow. The peaks at the top of the peaks are (hkl) Miller indices of bulk CeO_2_ with an FCC crystal structure.

The successful synthesis of the nanoparticles was further confirmed by X-ray diffraction (XRD). Before thermosgravimetric analysis (TGA), the nanoparticles exhibited very broad peaks, corresponding to a face-centered cubic (FCC) structure and reflecting ultrasmall particle diameters (Fig. 3a). However, after TGA up to 900 °C under airflow, they exhibited sharp peaks (Fig. 3b). All peaks could be assigned to the (hkl) Miller indices (111), (200), (220), (311), (222), (400), (331), (420), (422), and (511) of FCC CeO_2_, as indicated on the top of the peaks.^40,41^ The estimated cell constant (5.406 Å) was consistent with that (5.4113) of bulk CeO_2_ (JCPDS card no. 00-034-0394).^41^ Using Scherrer's formula,^42^ the diameters of the PAA- and PAAMA-coated ultrasmall CeO_2_ nanoparticles before TGA were estimated to be 1.06 and 1.07 nm, respectively, which were consistent with (or slightly less than) those observed in HRTEM images.

### Fourier transform-infrared (FT-IR) absorption spectra and TGA curves

**Fig. 4:**
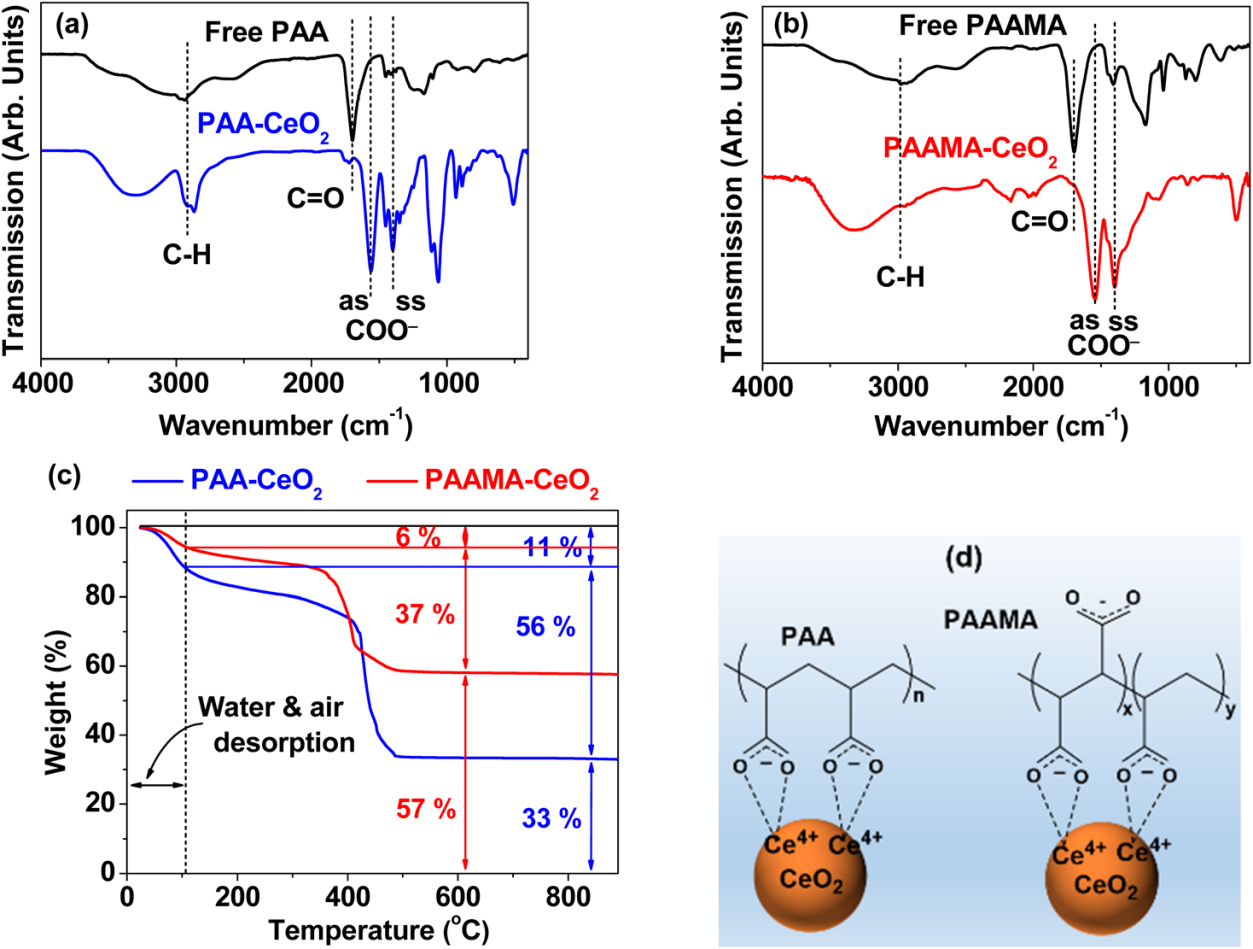
FT-IR absorption spectra of (a) free PAA and PAA-coated ultrasmall CeO_2_ nanoparticles and (b) free PAAMA and PAAMA-coated ultrasmall CeO_2_ nanoparticles. “as” and “ss” indicate the antisymmetric and symmetric stretching vibrations of COO^−^, respectively. (c) TGA curves of the PAA- and PAAMA-coated ultrasmall CeO_2_ nanoparticles under air flow. (d) Schematic of the coating structures of PAA and PAAMA polymers on the nanoparticle surfaces *via* electrostatic (*i.e.*, hard acid–base) bonding between the COO^−^ groups of the polymers and Ce^4+^ on the nanoparticle surfaces (the minor Ce^3+^ ions also exist on the nanoparticle surfaces, but only the major Ce^4+^ ions were displayed on the nanoparticle surfaces).

The surface coating of PAA and PAAMA on the nanoparticle surfaces was confirmed by FT-IR absorption spectra (Fig. 4a and b, respectively). The surface-coating amount was obtained from the TGA curves (Fig. 4c). As shown in Fig. 4a and b, C–H symmetric stretching vibration at ∼2930 cm^−1^, COO^−^ antisymmetric stretching vibration at ∼1550 cm^−1^, and COO^−^ symmetric stretching vibration at ∼1395 cm^−1^ confirmed the successful coating of PAA and PAAMA on the CeO_2_ nanoparticle surfaces. The red-shifts and splittings^43^ of the C=O symmetric stretching vibrations of the –COOH groups of free PAA and PAAMA at ∼1695 cm^−1^ into the symmetric and antisymmetric COO^−^ stretching vibrations in the FT-IR absorption spectra of the nanoparticle samples confirmed electrostatic (*i.e.*, hard acid–base) bonding^44^ between the COO^−^ groups of PAA and PAAMA and Ce^4+^ on the nanoparticle surfaces, as observed in other metallic oxide nanoparticles.^45,46^ Table S1† also summarizes the observed FT-IR absorption frequencies. The red-shifts of the COO^−^ antisymmetric and symmetric stretching vibrations from the C=O vibrations were ∼140 and ∼300 cm^−1^ (Table S1†), respectively, confirming the strong bonding. In addition, because PAA and PAAMA comprise many –COOH groups, they can bind to a nanoparticle *via* multiple bonds, as schematically drawn in Fig. 4d, consequently leading to the strong bonding of the polymer to the CeO_2_ nanoparticles and the long-term colloidal stability of the polymer-coated nanoparticles in aqueous media (*i.e.*, no precipitation after synthesis, >1.5 years).

The observed good colloidal stability confirmed that a sufficient amount of polymers should be coated on the CeO_2_ nanoparticle surfaces, which was confirmed from the TGA curves in Fig. 4c. The surface-coating amount (*S*) was estimated in wt% by measuring the mass losses after heating from ∼100 °C up to 900 °C because the initial mass drops (*i.e.*, 6% and 11%) up to ∼100 °C were attributed to the desorption of water and air. Grafting density (*σ*),^47,48^ defined as the average number of polymers coating a unit surface area of a nanoparticle, was obtained using the bulk density of CeO_2_ (7.132 g cm^−3^),^49^*d*_avg_ values estimated from HRTEM images, and aforementioned *S* values. The average number (*N*_polymer_) of polymers coating a nanoparticle was determined as the product of *σ* and nanoparticle surface area (=π*d*_avg_^2^). Table 1 summarizes the surface-coating results.

### *In vitro* cytotoxicity results

**Fig. 5:**
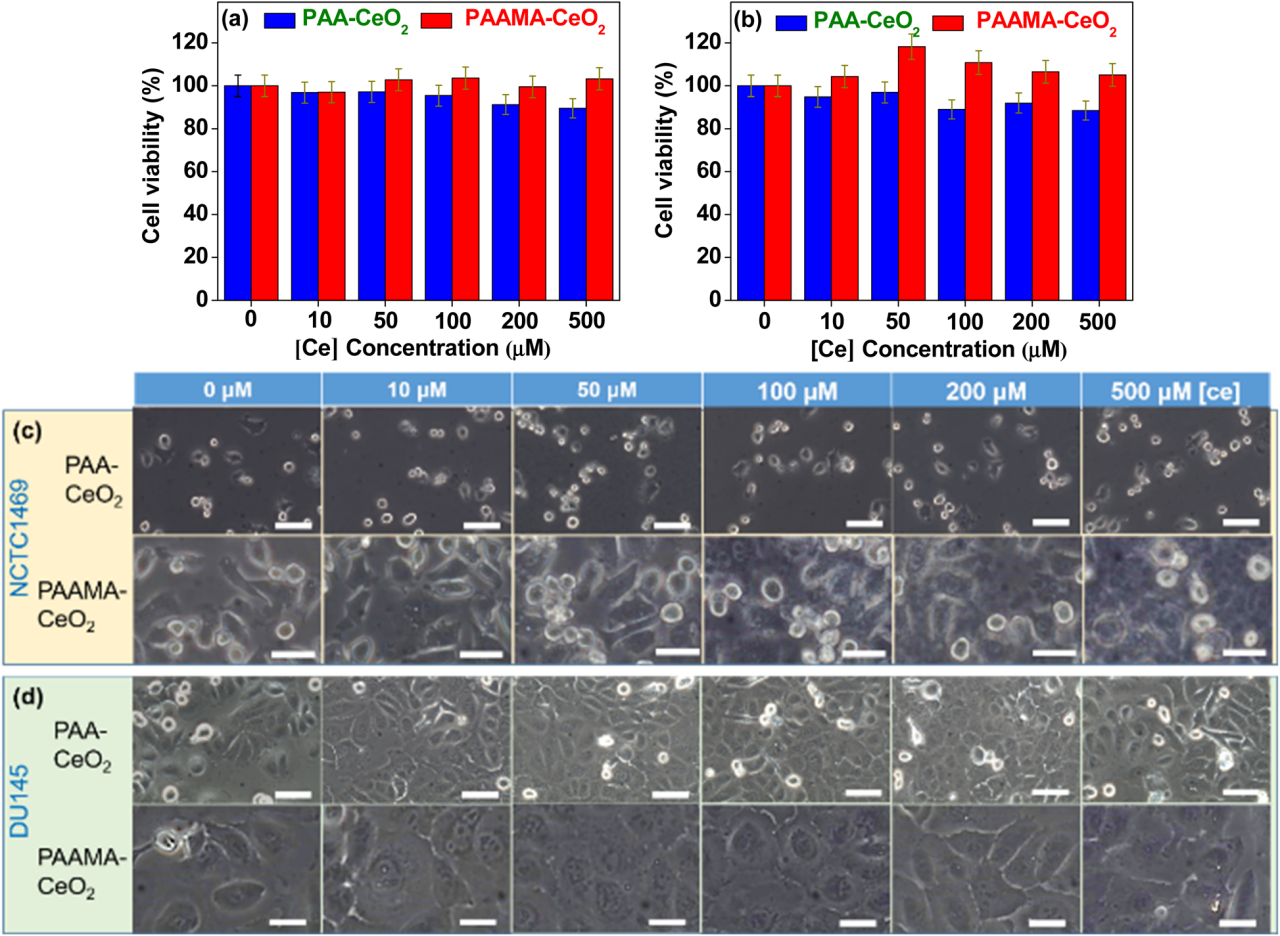
*In vitro* cell viability of (a) NCTC1469 and (b) DU145 cells and optical microscopy images of (c) NCTC1469 and (d) DU145 cells 48 h after incubation with the PAA- and PAAMA-coated ultrasmall CeO_2_ nanoparticles up to 500 μM [Ce]. Scale bar = 70 nm.

The PAA- and PAAMA-coated ultrasmall CeO_2_ nanoparticles exhibited very low *in vitro* cellular cytotoxicity (Fig. 5a and b), thereby demonstrating their suitability for biomedical applications. The high cell viability (>90%) of human prostate cancer (DU145) and normal mouse hepatocyte (NCTC1469) cells up to 500 μM [Ce] 48 h after incubation with nanoparticle samples was observed. Cell morphologies were examined using an optical microscope (Fig. 5c and d). The cell morphologies of the treated cells were similar to those of the control cells, which was consistent with the observed very low cellular cytotoxicity of the nanoparticles.

### Antioxidant effect

**Fig. 6:**
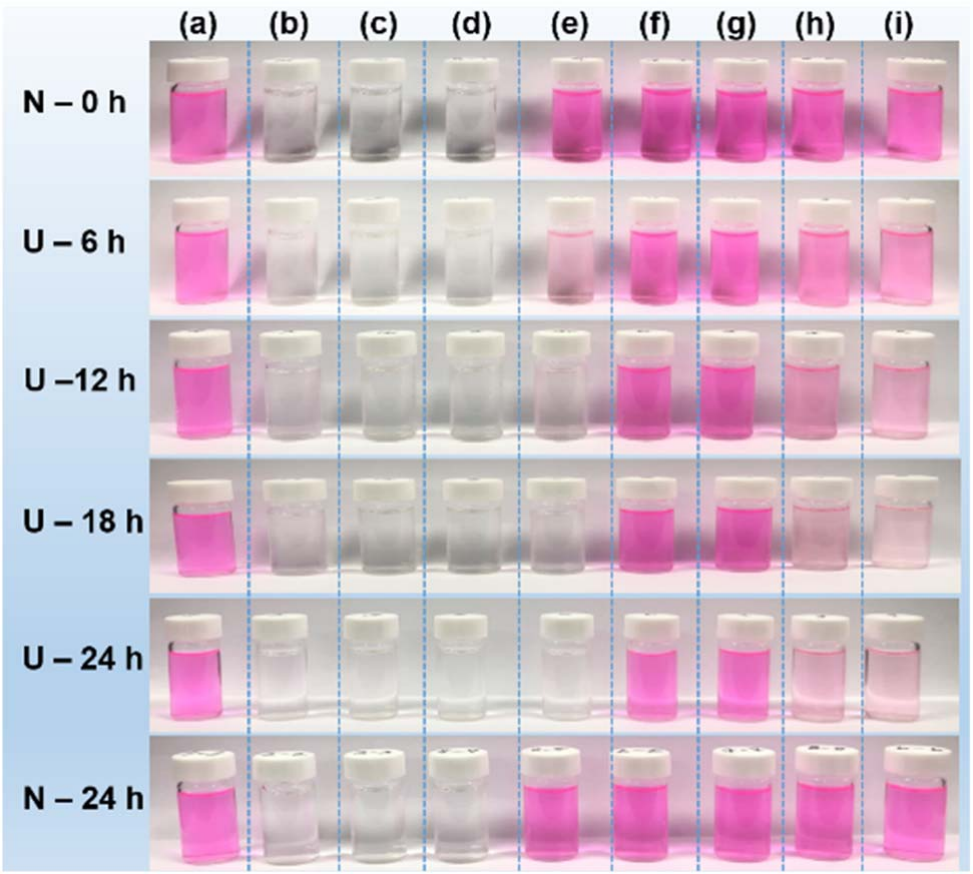
Photographs of various solutions up to 24 h: (a) 0.01 mM Rh B, (b) 0.1% H_2_O_2_, (c) PAA- and (d) PAAMA-coated ultrasmall CeO_2_ nanoparticles dispersed in aqueous media (0.1 mM [Ce]), (e) 0.01 mM Rh B + 0.05% H_2_O_2_, (f) 0.01 mM Rh B + PAA-coated ultrasmall CeO_2_ nanoparticles (0.05 mM [Ce]), (g) 0.01 mM Rh B + PAAMA-coated ultrasmall CeO_2_ nanoparticles (0.05 mM [Ce]), (h) 0.01 mM Rh B + 0.05% H_2_O_2_ + PAA-coated ultrasmall CeO_2_ nanoparticles (0.05 mM [Ce]), (i) 0.01 mM Rh B + 0.05% H_2_O_2_ + PAAMA-coated ultrasmall CeO_2_ nanoparticles (0.05 mM [Ce]). U = 365 nm UV irradiation (power = 15 W) and N = no UV irradiation.

**Fig. 7:**
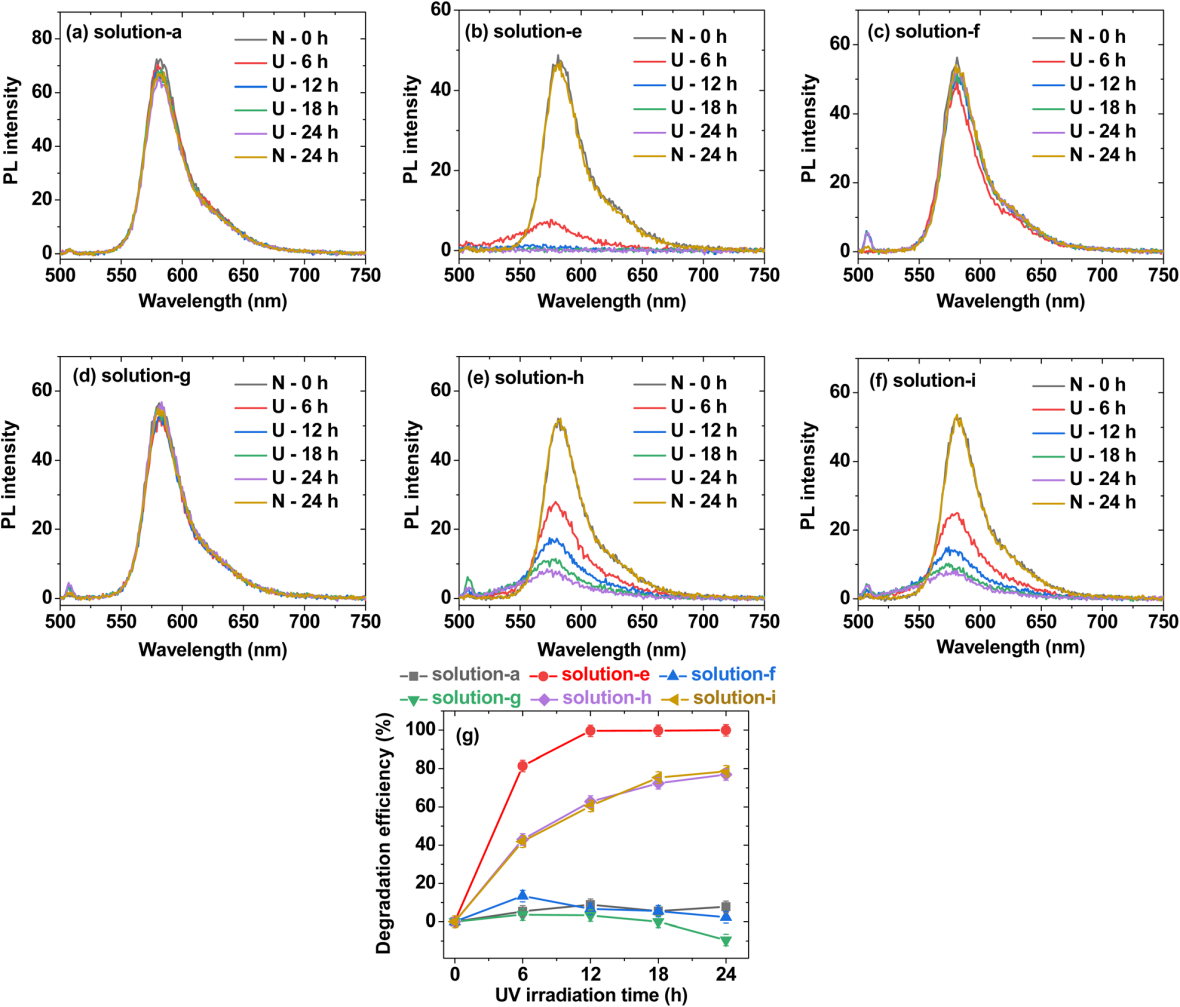
PL spectra of (a) solution-a (*i.e.*, 0.01 mM Rh B), (b) solution-e (*i.e.*, 0.01 mM Rh B + 0.05% H_2_O_2_), (c) solution-f {*i.e.*, 0.01 mM Rh B + PAA-coated ultrasmall CeO_2_ nanoparticles (0.05 mM [Ce])}, (d) solution-g {*i.e.*, 0.01 mM Rh B + PAAMA-coated ultrasmall CeO_2_ nanoparticles (0.05 mM [Ce])}, (e) solution-h {*i.e.*, 0.01 mM Rh B + 0.05% H_2_O_2_ + PAA-coated ultrasmall CeO_2_ nanoparticles (0.05 mM [Ce])}, (f) solution-i {*i.e.*, 0.01 mM Rh B + 0.05% H_2_O_2_ + PAAMA-coated ultrasmall CeO_2_ nanoparticles (0.05 mM [Ce])} in Fig. 6: U = 365 nm UV irradiation and N = no UV irradiation. (g) Plots of Rh B degradation efficiency (%) for solutions-a, -e, -f, -g, -h, and -i in Fig. 6.

To evaluate the antioxidant effect of the PAA- and PAAMA-coated ultrasmall CeO_2_ nanoparticles, the degradation of Rh B by oxidation with H_2_O_2_ was examined under 365 nm UV irradiation in the presence and absence of the polymer-coated nanoparticles. Decolorization photographs and photoluminescence (PL) spectra of nine solutions prepared in aqueous media were measured as a function of time under UV irradiation: (a) 0.01 mM Rh B, (b) 0.1% H_2_O_2_, (c) PAA- and (d) PAAMA-coated ultrasmall CeO_2_ nanoparticles (0.1 mM [Ce]), (e) 0.01 mM Rh B + 0.05% H_2_O_2_, (f) 0.01 mM Rh B + PAA-coated ultrasmall CeO_2_ nanoparticles (0.05 mM [Ce]), (g) 0.01 mM Rh B + PAAMA-coated ultrasmall CeO_2_ nanoparticles (0.05 mM [Ce]), (h) 0.01 mM Rh B + 0.05% H_2_O_2_ + PAA-coated ultrasmall CeO_2_ nanoparticles (0.05 mM [Ce]), (i) 0.01 mM Rh B + 0.05% H_2_O_2_ + PAAMA-coated ultrasmall CeO_2_ nanoparticles (0.05 mM [Ce]). The solution photographs (Fig. 6) and PL spectra (Fig. 7) were measured at intervals of 6 h up to 24 h. Rh B, including other organic dyes, very slowly decomposes under UV irradiation and its decomposition rate depends on the UV irradiation intensity.^50–54^ However, Rh B undergoes rapid decomposition in the presence of the oxidizing agent H_2_O_2_ under UV irradiation according to the following oxidation reaction,^55^Rh B + H_2_O_2_ + UV → Rh B + ˙OH → NO_3_^−^ + NH_4_^+^ + CH_4_ + CO_2_ + H_2_O

A similar oxidation reaction of Rh B was observed in the Rh B/H_2_O_2_/hydroxylamine (HA) system in which HA reacted with H_2_O_2_ to generate hydroxyl radical (˙OH) to decompose Rh B.^55^ As shown in Fig. 6, solution-a exhibited an unnoticeable degradation of pink color up to 24 h, indicating that Rh B negligibly decomposed without H_2_O_2_ regardless of 365 nm UV irradiation (power = 15 W). Solutions-f and -g also exhibited unnoticeable pink color degradation up to 24 h, indicating that Rh B did not undergo decomposition by the PAA- and PAAMA-coated ultrasmall CeO_2_ nanoparticles regardless of the UV irradiation. Solutions-b, -c, and -d were transparent (*i.e.*, no color) because of the absence of Rh B in solutions, indicating that the pink color was solely attributed to Rh B, and not H_2_O_2_ and PAA- and PAAMA-coated ultrasmall CeO_2_ nanoparticles. In the case of solution-e, Rh B rapidly degraded (*i.e.*, rapid pink color degradation) due to the aforementioned oxidation reaction of Rh B with H_2_O_2_ under the UV irradiation. By contrast, in solutions-h and -I, the retarded degradation of Rh B (*i.e.*, retarded pink color degradation) was observed due to the antioxidant effect of the PAA- and PAAMA-coated ultrasmall CeO_2_ nanoparticles because CeO_2_ removed H_2_O_2_ according to the following reaction (therefore, the oxidation reaction of Rh B with H_2_O_2_ under UV irradiation was retarded by CeO_2_),^56,57^Ce^4+^ + H_2_O_2_ → Ce^3+^ + H^+^ + HO_2_Ce^4+^ + HO_2_ → Ce^3+^ + H^+^ + O_2_

Therefore, the net antioxidant reaction was as follows:2Ce^4+^ + H_2_O_4_ → 2Ce^3+^ + 2H^+^ + O_2_.

The antioxidant effects of the PAA- and PAAMA-coated ultrasmall CeO_2_ nanoparticles were quantitatively investigated by recording PL spectra (Fig. 7a–f). Solutions-a, -f, and -g exhibited an unnoticeable PL intensity drop with time up to 24 h (Fig. 7a, c and d, respectively), which was consistent with the observation of unnoticeable pink color degradation in the solution photographs in Fig. 6a, f and g, respectively. The PL spectra of solutions-b, -c, and -d were not measured because Rh B was absent in the solutions. Solution-e exhibited a rapid drop in the PL intensity with time (Fig. 7b), whereas solutions-h and -i containing nanoparticles exhibited a delayed drop in the PL intensity (Fig. 7e and f, respectively), confirming the antioxidant effect of the nanoparticles. To quantitatively evaluate the degradation efficiency (%) of Rh B with time, defined as 100 (*I*_0_ − *I*_*t*_)/*I*_0_, where *I*_*t*_ is the PL intensity at time *t*, it was plotted as a function of time in Fig. 7g. Solutions-a, -f, and -g exhibited a negligible degradation efficiency of Rh B overtime. Solution-e rapidly exhibited ∼100% degradation efficiency of Rh B at 12 h, whereas solutions-h and -i exhibited only ∼78% degradation efficiency of Rh B at 24 h due to the antioxidant effect of the nanoparticles. This result confirmed the antioxidant effect of the PAA- and PAAMA-coated CeO_2_ nanoparticles; therefore, these nanoparticles exhibited potential as radioprotective or theranostic X-ray contrast agents by removing ROS (*i.e.*, H_2_O_2_ and ˙OH) produced by X-rays during X-ray scan.

### X-ray attenuation: phantom images

**Fig. 8:**
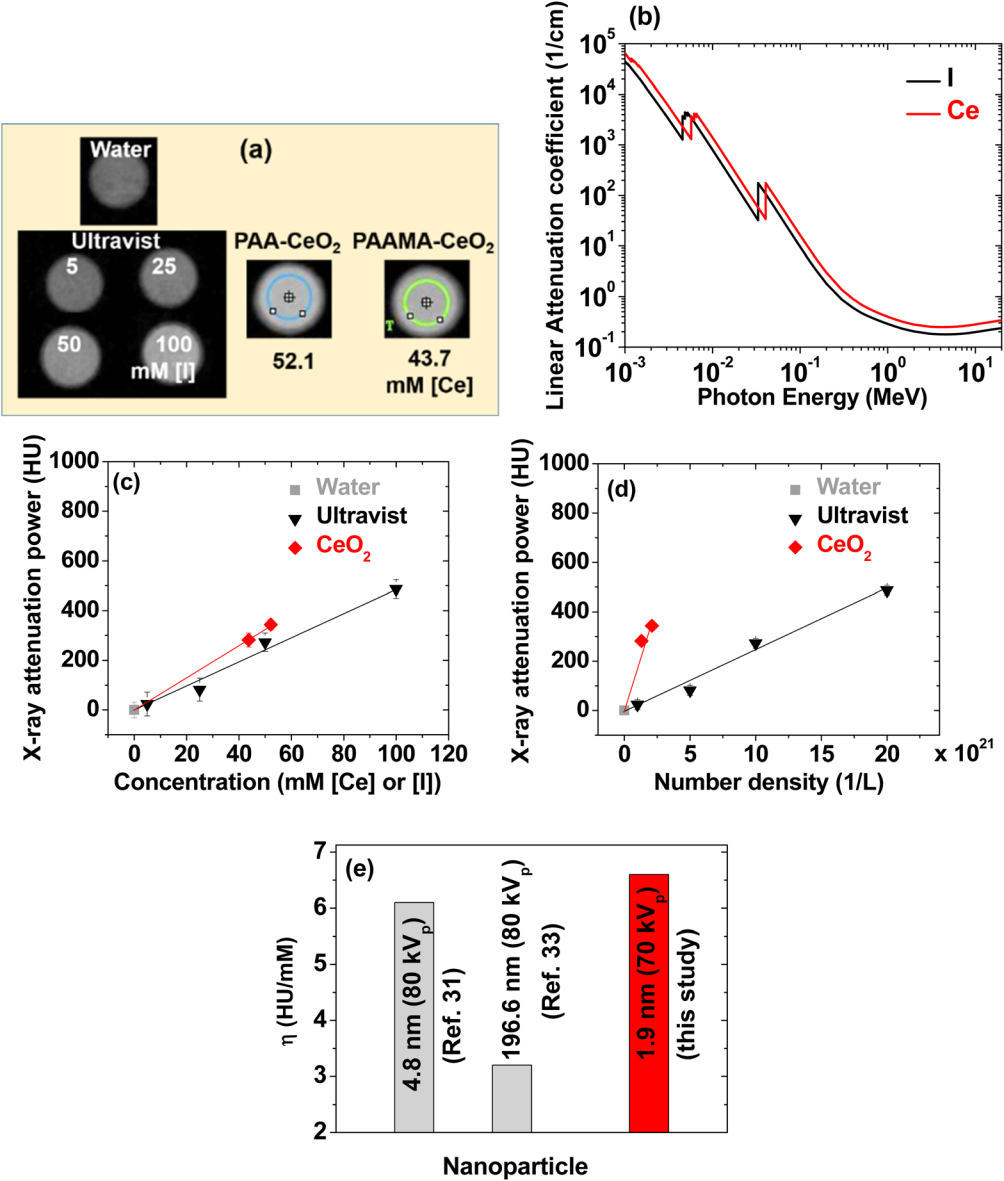
(a) X-ray phantom images of Ultravist and PAA- and PAAMA-coated ultrasmall CeO_2_ nanoparticles dispersed in aqueous media at an X-ray source voltage of 70 kV_p_. (b) Plot of the linear attenuation coefficients of Ce and I *versus* radiation photon energy. Plots of the X-ray attenuation as a function of the (c) atomic concentrations of [Ce] and [I] and (d) number density of the nanoparticles and Ultravist: slopes of the dotted lines correspond to X-ray attenuation efficiencies (*η*). (e) Comparison of *η* values: dextran-coated CeO_2_ nanoparticles (*d* = 4.8 nm, 80 kV_p_),^35^ porous Ce_2_(CO_3_)_2_O·H_2_O nanoparticles (*d* = 196.6 nm, 80 kV_p_),^39^ and polymer-coated ultrasmall CeO_2_ nanoparticles [*d* = (1.8 + 2.0)/2 = 1.9 nm, 70 kV_p_] (this study). Water: 0 HU.

The contrasts of the PAA- and PAAMA-coated ultrasmall CeO_2_ nanoparticles in the X-ray phantom images were brighter than those of a commercial molecular iodine(i) contrast agent Ultravist at similar atomic concentrations of [Ce] and [I] (Fig. 8a), demonstrating that the PAA- and PAAMA-coated ultrasmall CeO_2_ nanoparticles were superior than Ultravist. This result was attributed to the higher linear X-ray attenuation coefficient of Ce than that of I (Fig. 8b).^22^ To quantitatively discuss this result, X-ray attenuation estimated from X-ray phantom images was plotted as a function of the atomic concentration. The X-ray attenuation of the PAA- and PAAMA-coated ultrasmall CeO_2_ nanoparticles was greater than that of Ultravist at the same atomic concentration of [Ce] and [I] at 70 kV_p_ (Fig. 8c). In addition, Fig. 8d shows the X-ray attenuation of the nanoparticles as a function of the number density: the X-ray attenuation at the same number density was greater than that observed at the same atomic concentration: therefore, nanoparticle contrast agents can provide considerably higher contrast enhancement than molecular agents at the same number density, making the nanoparticle contrast agents superior than molecular contrast agents. The number density was estimated by multiplying the molar atomic concentration with 6.02 × 10^23^/*N*_atom_, where *N*_atom_ is the number of X-ray attenuating atoms per molecule or nanoparticle; *N*_atom_ is three for Ultravist, and ∼(1/3) (*d*_avg_/*h*)^3^ = 150 and 205 for PAA- and PAAMA-coated ultrasmall CeO_2_ nanoparticles,^58^ respectively; in the above formula, *h* represents the average ionic diameter of the atoms per chemical formula [=2{0.101 (Ce^4+^) + 2 × 0.126 (O^2−^)}/3 = 0.235 nm].^59^

As a key parameter for comparing materials as X-ray contrast agents, the X-ray attenuation efficiency (*η*), defined as the X-ray attenuation per molar concentration [Hounsfield units (HU)/mM] or per number density [HU/(1/L)], was estimated from the slopes in Fig. 8c and d, respectively. Table 2 summarizes the results. The *η* values of the nanoparticles were 1.3 and 68 times greater than those of Ultravist in terms of the molar atomic concentration and number density, respectively. In addition, the *η* value estimated herein was greater than those^35,39^ of larger CeO_2_ nanoparticles (Fig. 8e). This result was attributed to the particle size effect, *i.e.*, smaller nanoparticles can attenuate X-rays more effectively than larger nanoparticles because of the exponential decay of X-rays along the penetration depth. Therefore, the results obtained herein revealed that the PAA- and PAAMA-coated ultrasmall CeO_2_ nanoparticles demonstrate promise as highly sensitive X-ray contrast agents.

**Table 2:** Summary of the observed X-ray attenuation properties of Ultravist and PAA- and PAAMA-coated ultrasmall CeO_2_ nanoparticles dispersed in aqueous media at 70 kV_p_

Chemical	*N*_atom_	Concentration (mM [Ce] or [I])	Number density (1/L) × 10^20^	X-ray attenuation (HU)	X-ray attenuation efficiency (*η*)	
				70 kV_p_	(HU/mM)	[HU/(1/L)] × 10^−19^
PAA-CeO_2_	150	52.1	2.1	344	6.6	16.9
PAAMA-CeO_2_	205	43.7	1.3	282		
Ultravist	3	100	200.7	487	5.0	0.25
	3	50	100.3	273		
	3	25	50.2	82		
	3	5	10.0	24		

### *In vivo* CT images

**Fig. 9:**
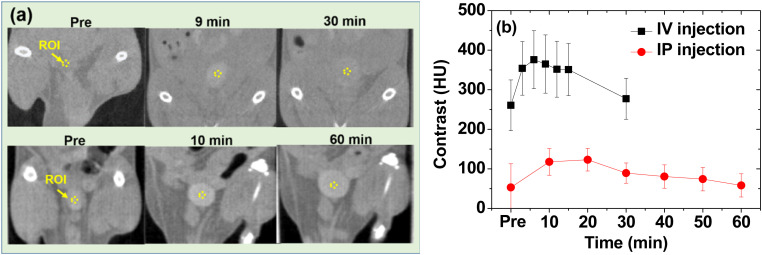
(a) *In vivo* CT images of the mice bladder before and after intravenous (IV) and intraperitoneal (IP) injections of an aqueous suspension sample of PAA-coated ultrasmall CeO_2_ nanoparticles at 70 kV_p_. The dotted circles at the bladder indicate the region of interest (ROI). (b) Contrast plots of the SNR-ROI of the bladder as a function of time.

The potential of the nanoparticles as X-ray contrast agents was further confirmed *in vivo* using the PAA-coated ultrasmall CeO_2_ nanoparticles. The nanoparticles dispersed in aqueous media were injected *via* two routes: intravenously (IV) *via* the mice tails and intraperitoneally (IP). The CT images were recorded before and after injection using an injection dose of ∼0.1 mmol Ce per kg, which was less than that (>1 mmol I per kg)^6,20^ of the iodine contrast agents. Positive contrast enhancement was observed in the mice bladder after IV and IP injections even at an injection dose of ∼10 times less than those of iodine contrast agents (Fig. 9a). The contrasts were quantitatively shown in Fig. 9b by plotting the signal-to-noise ratio (SNR) of a region of interest (ROI) at the bladder as a function of time. Compared with the IP injection, the IV injection exhibited a more rapid SNR increase and drop due to the faster excretion of the nanoparticles after the IV injection than that after the IP injection.^60,61^ This *in vivo* result confirmed that the PAA- and PAAMA-coated ultrasmall CeO_2_ nanoparticles demonstrate potential as CT contrast agents.

## Experimental

### Synthesis of polymer-coated ultrasmall CeO_2_ nanoparticles (polymer = PAA and PAAMA)

The schematic of the one-pot polyol synthesis^58,62^ is shown in Fig. S1,† and details of the synthesis are provided in ESI.† In this method, triethylene glycol (TEG) as solvent suppressed the particle size growth, leading to TEG-coated ultrasmall CeO_2_ nanoparticles. Then, TEG was replaced with PAA (or PAAMA) because –COOH groups of the PAA (or PAAMA) can more strongly bind to the CeO_2_ nanoparticles than –OH group of the TEG.

### General characterization

The synthesized nanoparticles were characterized as described in detail in previous studies.^58,62^ The Ce concentration of the nanoparticle suspension in aqueous media was measured by inductively coupled plasma-atomic emission spectrometry (Avio500, PerkinElmer, Waltham, MA, USA). The particle diameters of the PAA- and PAAMA-coated ultrasmall CeO_2_ nanoparticles were estimated by HRTEM (Titan G2 ChemiSTEM CS Probe, FEI, Hillsboro, OR, USA) operating at an accelerating voltage of 200 kV. Hydrodynamic diameters (a) and zeta potentials (*ζ*) were measured using a particle size analyzer (Zetasizer Nano ZS, Malvern Panalytical, Malvern, UK) with diluted samples (∼0.1 mM [Ce]). The crystal structure of the powder samples before and after TGA was measured using a multipurpose powder XRD spectrometer (X-PERT PRO MRD, Philips, Eindhoven, The Netherlands) with unfiltered CuKa (*λ* = 1.54184 Å) radiation. The surface coating of the polymers on nanoparticle surfaces was confirmed by recording FT-IR absorption spectra (Galaxy 7020A, Mattson Instruments, Inc., Madison, WI, USA) using dried powder samples pelletized in KBr. The surface-coating amount of polymers on nanoparticle surfaces was estimated by recording TGA curves (SDT-Q600, TA Instruments, New Castle, DE, USA) between room temperature (∼20 °C) and 900 °C under airflow. The antioxidant effect was measured by recording PL spectra (Cary Eclipse, Agilent Technologies) of various solutions of Rh B and H_2_O_2_ in aqueous media under 365 nm UV irradiation (15 W, Vilber Lourmat, Cedex 1, France) in the presence and absence of the nanoparticle samples.

### *In vitro* cell viability measurements

The *in vitro* cytotoxicity of polymer-coated ultrasmall CeO_2_ nanoparticles was measured using the DU145 and NCTC1469 cell lines. A cell viability assay kit (CellTiter-Glo, Promega, Madison, WI, USA) was used. The adenosine triphosphate content was measured using a luminometer (Victor 3, PerkinElmer, Waltham, MA, USA). The cells were seeded onto a 24-well cell culture plate (5 × 10^4^ cell density, 500 μL cells per well) and incubated for 24 h (5% CO_2_, 37 °C). Five test nanoparticle solutions (10, 50, 100, 200, and 500 μM [Ce], respectively) in a sterile phosphate buffer saline solution (PBS) were prepared by diluting the original concentrated nanoparticle suspension (∼50 mM [Ce]) with PBS. Approximately 2 μL of each test solution was added to the cells and the treated cells were incubated for 48 h. The cell viabilities were measured thrice, and the average values were normalized with respect to those of the control cells (*i.e.*, untreated cells with nanoparticle samples).

### X-ray phantom image measurements

X-ray attenuation was estimated by measuring X-ray phantom images using a micro-CT scanner (Inveon, Siemens Healthcare, Erlangen, Germany) at an X-ray source voltage of 70 kV_p_, an X-ray source current of 280 μA, and an imaging time per frame of 300 ms. It was estimated in HU with respect to that of water with 0.0 HU using the formula HU = 1000 (*μ*_sample_ − *μ*_water_)/*μ*_water_, where *μ* is the measured linear attenuation coefficient of the material from the phantom images.

### Animal studies

All animal procedures were performed in accordance with the Guidelines for Care and Use of Laboratory Animals of Kyungpook National University (KNU) (IV injection experiment) and Korea Institute of Radiological & Medical Science (KIRAMS) (IP injection experiment) and approved by the Animal Ethics Committee of KNU and KIRAMS (permission no. 2022-0345 and kirams2023-0012, respectively).

### *In vivo* CT image measurements

Female ICR mice (ICR = Institute of Cancer Research, USA) with a weight of ∼40 g were injected with 0.1 mmol Ce per kg and used for imaging. For imaging, the mice were anesthetized using 1.5% isoflurane in oxygen, and measurements were conducted before and after IV injection with the PAA-coated ultrasmall CeO_2_ nanoparticles dispersed in aqueous media into the mice tails under the following conditions: number of mice (*N*) = 2, X-ray source voltage = 70 kV_p_, X-ray source current = 280 μA, imaging time per frame = 1700 ms, thickness = 0.148 nm, and resolution = 512 × 512. The measurements were also conducted before and after IP injection (200 μL). After measurements, the mice were revived from anesthesia and placed in a cage with free access to food and water.

## Conclusions

Hydrophilic and biocompatible PAA- and PAAMA-coated ultrasmall CeO_2_ nanoparticles (*d*_avg_ values of 1.8 and 2.0 nm, respectively, the smallest size reported thus far) were synthesized using the one-pot polyol method.

(1) The nanoparticles exhibited excellent colloidal stability (*i.e.*, no precipitation after synthesis, >1.5 years) and low cellular toxicity (*i.e.*, >90% cell viability).

(2) Their X-ray attenuation efficiency was 1.3 times greater than that of Ultravist. Furthermore, it was greater than those of various large CeO_2_ nanoparticles reported previously.

(3) They exhibited an antioxidant effect for the removal of H_2_O_2_.

(4) The results from in *in vivo* mice experiments confirmed that the nanoparticles exhibited contrast enhancement after IV and IP injections. All these results suggested that PAA- and PAAMA-coated ultrasmall CeO_2_ nanoparticles are highly sensitive X-ray contrast agents with antioxidant effects.

## Author contributions

Abdullah Khamis Ali Al Saidi: experimental and draft writing. Adibehalsadat Ghazanfari: experimental. Ahrum Baek: IV injection CT image acquisition. Tirusew Tegafaw, Mohammad Yaseen Ahmad, Dejun Zhao and Ying Liu: data curation and methodology. Ji-ung Yang and Ji Ae Park: IP injection CT image acquisition. Byeong Woo Yang: validation. Kwon Seok Chae: cell viability assay. Sung-Wook Nam: funding. Yongmin Chang and Gang Ho Lee: funding, supervision and writing.

## Conflicts of interest

There are no conflicts to declare.

## Supplementary Material

RA-014-D3RA08372A-s001
