# Supplementary material for: Ultrasmall cerium oxide nanoparticles as highly sensitive X-ray contrast agents and their antioxidant effect
Source: RSC Adv. 2024 Jan 23;14(6):3647–58. doi: 10.1039/d3ra08372a (PMC10805080; doi:10.1039/d3ra08372a)
Supplement: RA-014-D3RA08372A-s001 [file RA-014-D3RA08372A-s001.pdf]

Electronic Supplementary Material (ESI) for RSC Advances.
This journal is © The Royal Society of Chemistry 2024

Supplementary Information

Ultrasmall cerium oxide nanoparticles as highly sensitive X-ray contrast agents and their antioxidant effect

Abdullah Khamis Ali Al Saidi,[a] Adibehalsadat Ghazanfari,[a] Ahrum Baek,[b] Tirusew Tegafaw,[a] Mohammad Yaseen Ahmad,[a] Dejun Zhao,[a] Ying Liu,[a] Ji-ung Yang,[c] Ji Ae Park,[c] Byeong Woo Yang,[d] Kwon Seok Chae,[e] Sung-Wook Nam,[f] Yongmin Chang[*f] and Gang Ho Lee[*a]

[a]Department of Chemistry, College of Natural Sciences, Kyungpook National University, Taegu 41566, South Korea

[b]Institute of Biomedical Engineering, School of Medicine, Kyungpook National University, Taegu 41944, South Korea

[c]Division of Applied RI, Korea Institute of Radiological & Medical Sciences, Seoul, 01812, South Korea

[d]Theranocure, Medlifescience Bldg. 1, Chilgok, Bukgu,Taegu 41405, South Korea

[e]Department of Biology Education, Teachers' College, Kyungpook National University, Taegu 41566, South Korea

[f]Department of Molecular Medicine, School of Medicine, Kyungpook National University, Taegu 41944, South Korea

*Corresponding authors: ghlee@mail.knu.ac.kr (G.H.L.); ychang@knu.ac.kr (Y.C.)

1. Synthesis

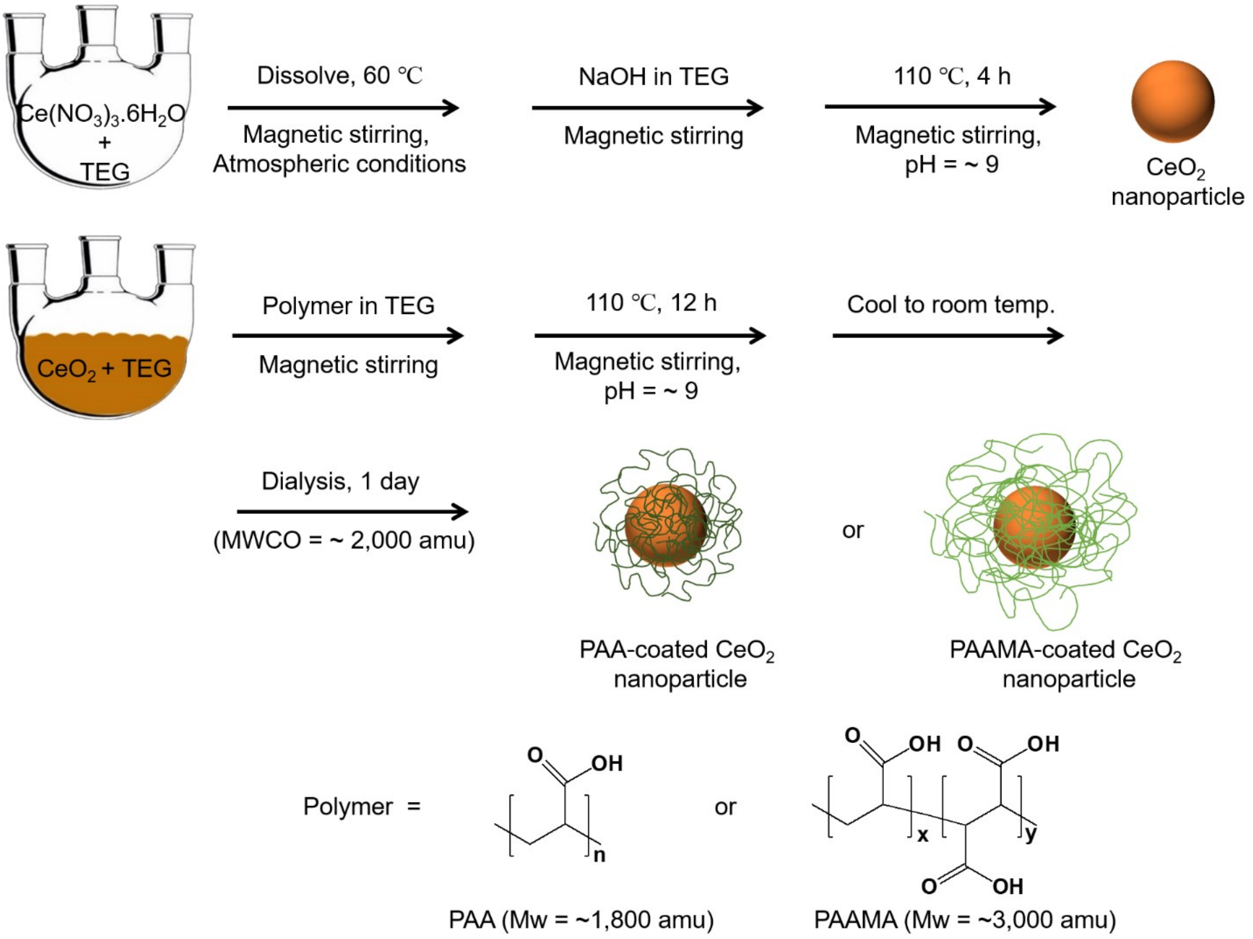

Fig. S1 One-pot polyol synthesis of polymer-coated ultrasmall CeO_2 nanoparticles: polymer = poly(acrylic acid) (PAA) and poly(acrylic acid-co-maleic acid) (PAAMA).

1.1 Chemicals

Chemicals used in the synthesis such as cerium (III) nitrate hexahydrate [$Ce(NO_3)_3.6H_2O$, 99%], sodium hydroxide (NaOH, > 99.99%), hydrogen peroxide solution (H_2O_2, 50 wt. % in H_2O), rhodamine B (Rh B; $C_{28}H_{31}ClN_2O_3$, 97%), PAA [$(C_3H_4O_2)_n$, 99%, Mw = ~1,800 amu], PAAMA [$(C_3H_4O_2)_x(C_4H_4O_4)_y$, 99%, Mw = ~3,000 amu], and triethylene glycol (TEG;

$C_6H_{14}O_4$, 99%), potassium bromide (KBr, ≥99%), and dialysis tube [molecular weight cut off (MWCO) = 2,000 amu] were purchased from Sigma-Aldrich (St. Louis, MO, USA). Ethanol (C_2H_6O, 99%) was purchased from Duksan (Ansan, South Korea). All chemicals were used as received.

1.2 Synthesis of polymer-coated ultrasmall CeO_2 nanoparticles (polymer = PAA and PAAMA)

A schematic one-pot polyol synthesis is shown in Fig. S1. A precursor solution is prepared by dissolving 2 mmol of $Ce(NO_3)_3.6H_2O$ in 20 mL of TEG in a 100-mL three-necked round bottom flask via magnetic stirring at 60 °C for 30 min under atmospheric conditions. In a separate beaker, 10 mmol of NaOH in 20 mL of TEG was prepared via magnetic stirring at 60 °C for 3 h under atmospheric conditions. In another beaker, 0.5 mmol of polymer (i.e., PAA or PAAMA) in 10 mL of TEG was prepared via magnetic stirring at room temperature for 3 h under atmospheric conditions. The NaOH solution was slowly added to the precursor solution until the pH of the mixed solution reached 9~10. Then the mixed solution was slowly heated to 110 °C with magnetic stirring for 4 h. The polymer solution was slowly added to the mixed solution and magnetically stirred under the same conditions for 12 h. For temperature control, the three-necked round bottom flask was suspended in a silicone oil bath placed on a hot plate. The product solution was cooled to room temperature, poured into a 1 L beaker, diluted with 500 mL of ethanol, magnetically stirred for 15 min, and then kept in a refrigerator (4 °C) for a few days until polymer-coated CeO_2 nanoparticles settled to the bottom of the beaker. The top transparent solution was decanted and the remaining product

nanoparticle solution was washed with ethanol again using the same process thrice. To remove ethanol from the nanoparticles, 400 mL of triple-distilled water was added and the solution was reduced to approximately 50 mL via rotary evaporation. The product solution was dialyzed using a dialysis tube (MWCO = 2,000 amu) against 1 L of triple-distilled water for one day with magnetic stirring under atmospheric conditions: the triple-distilled water was replaced with fresh water thrice during dialysis.

2. Tyndall effect

The colloidal dispersion was confirmed by Tyndall effect (Fig. S2).

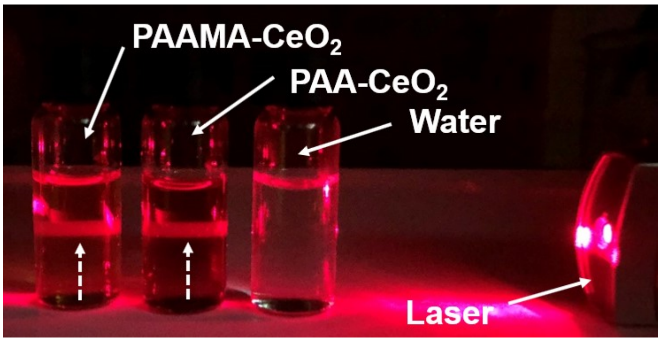

Fig. S2 Laser light scattering (indicated with vertical dotted arrows) of the aqueous nanoparticle suspension samples, confirming the colloidal dispersion, but such scattering in triple-distilled water was not observed.

3. High-resolution transmission electron microscope images

3.1 PAA-coated ultrasmall CeO_2 nanoparticles

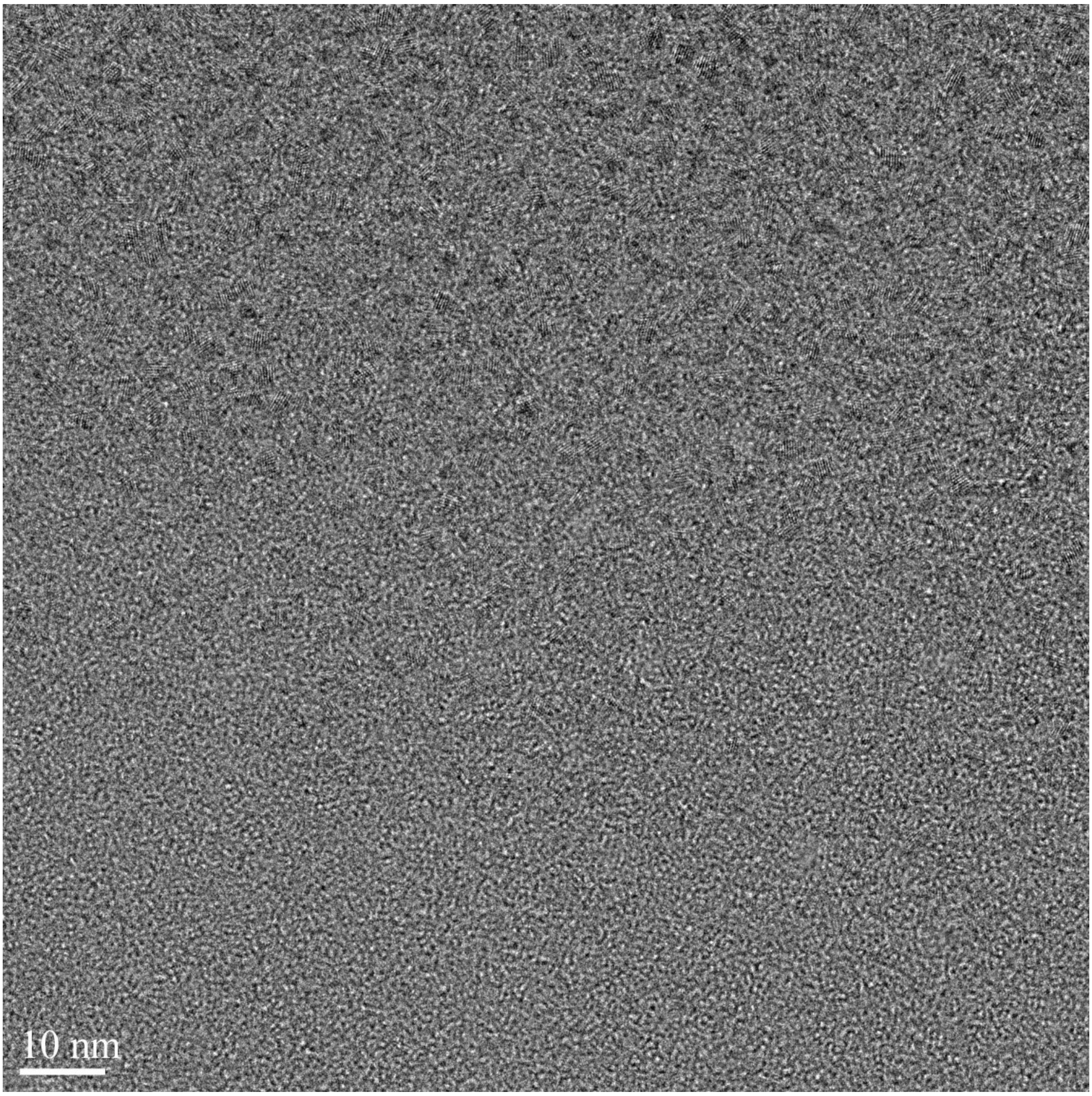
10 nm

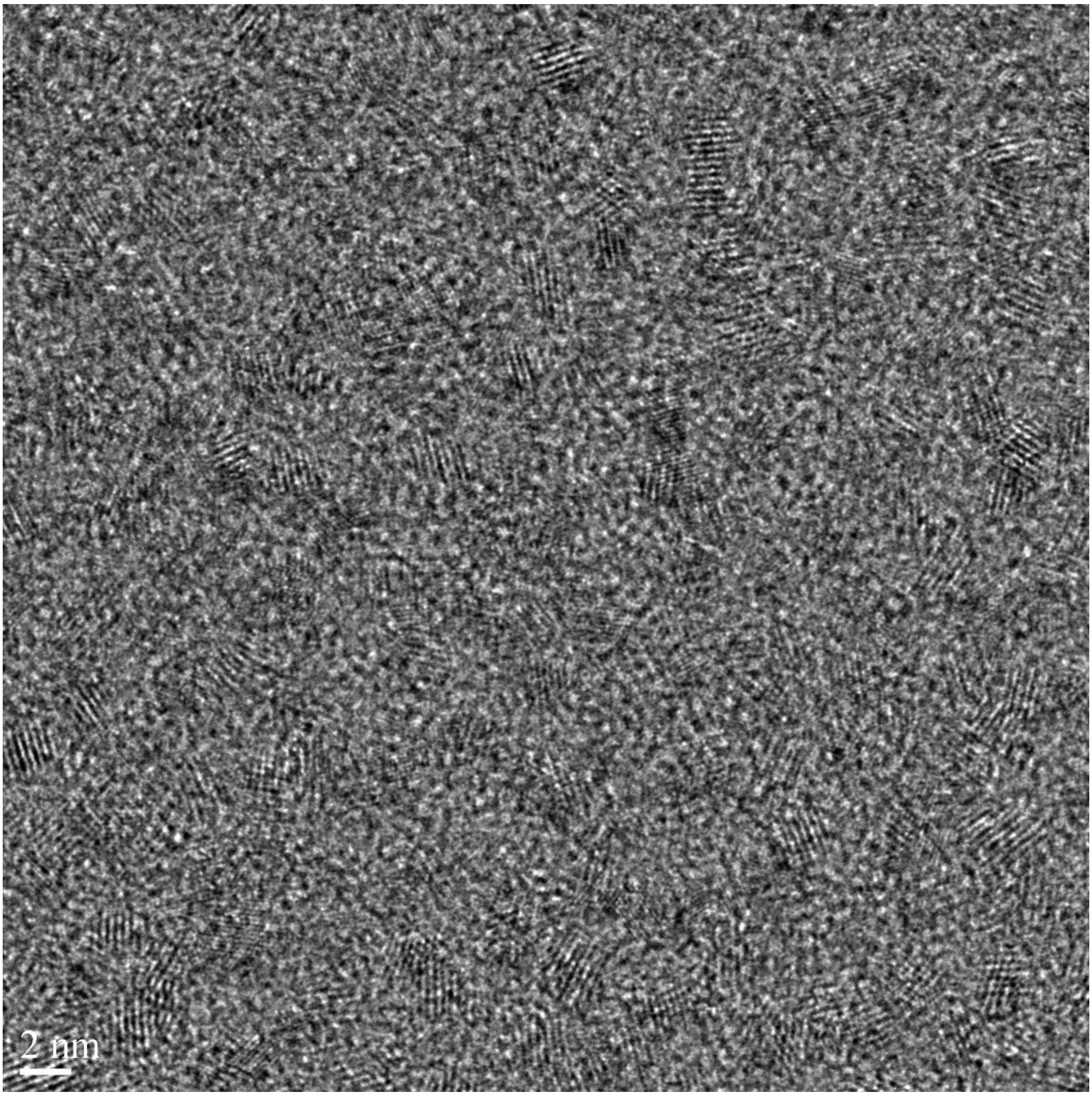
2 nm

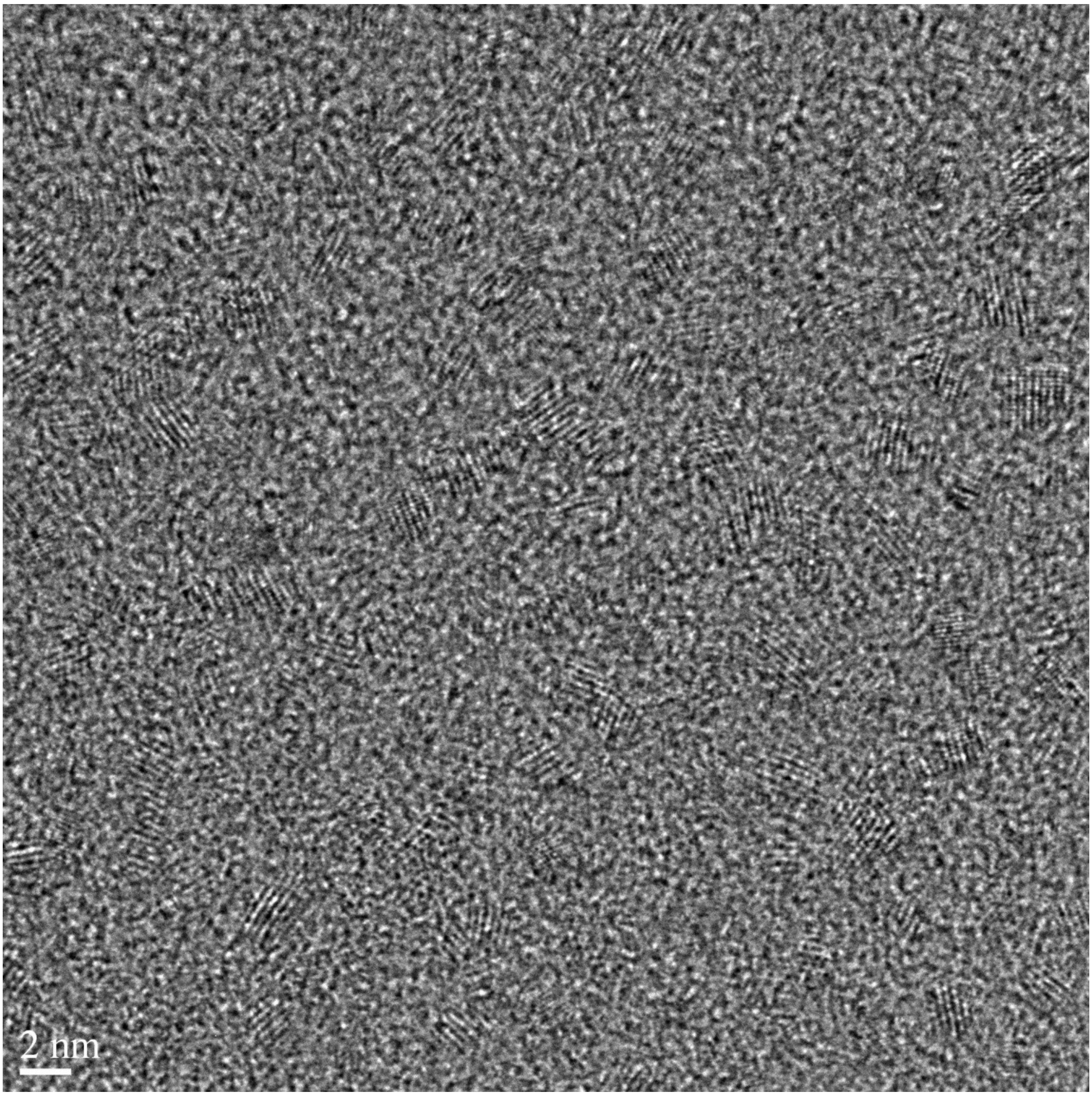

Fig. S3 HRTEM images of PAA-coated ultrasmall CeO_2 nanoparticles.

3.2 PAAMA-coated ultrasmall CeO_2 nanoparticles

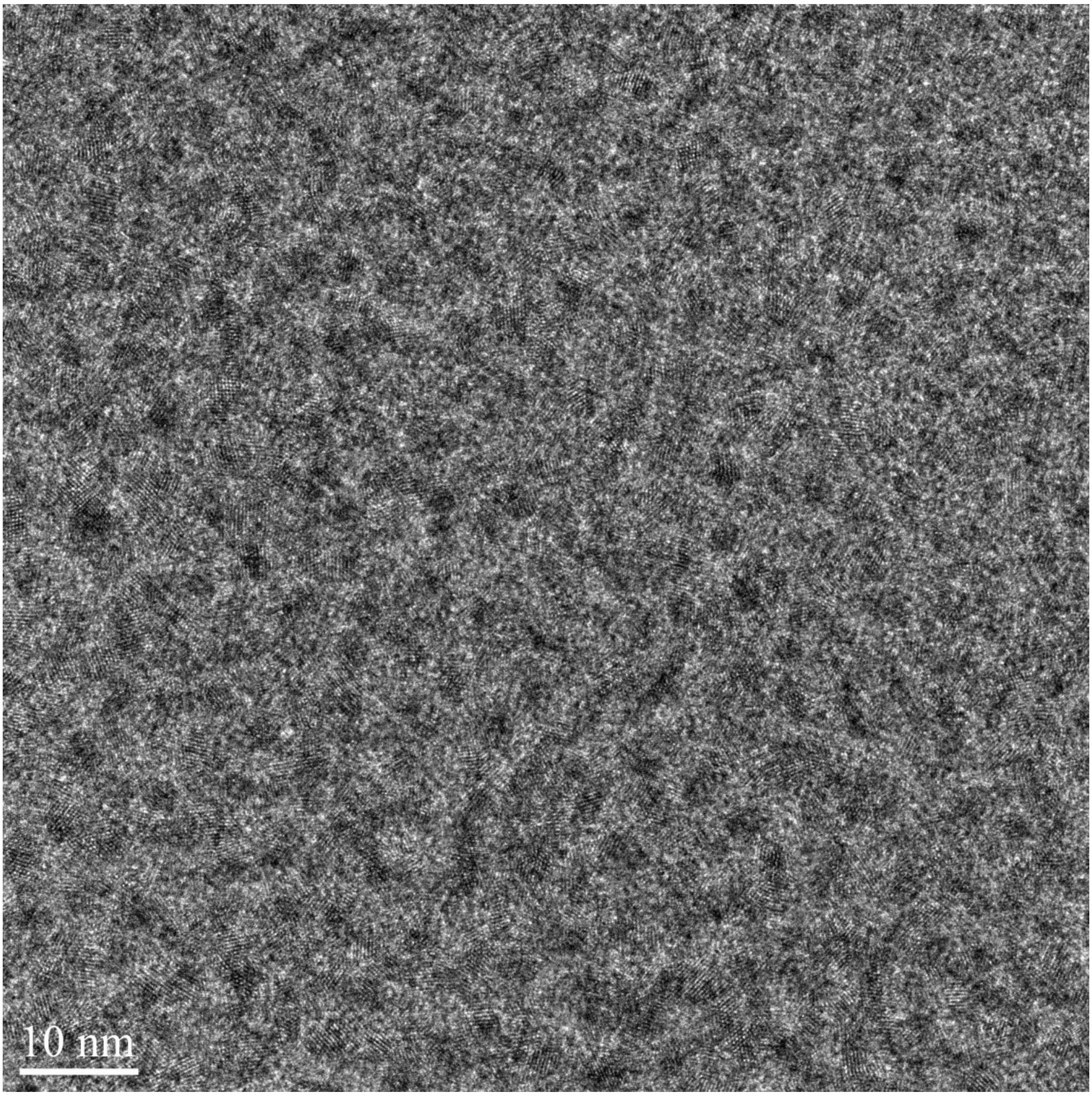
10 nm

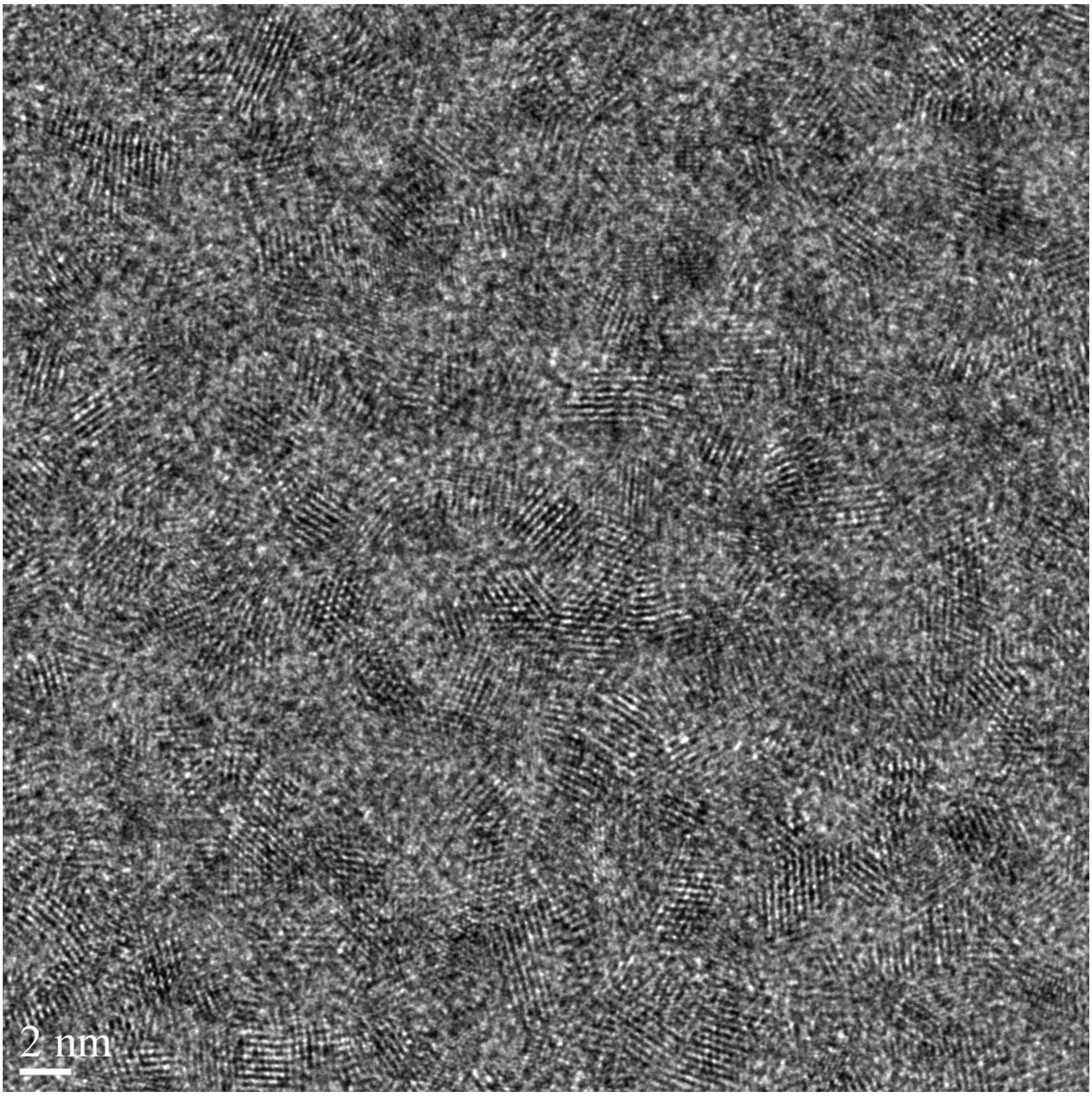
2 nm

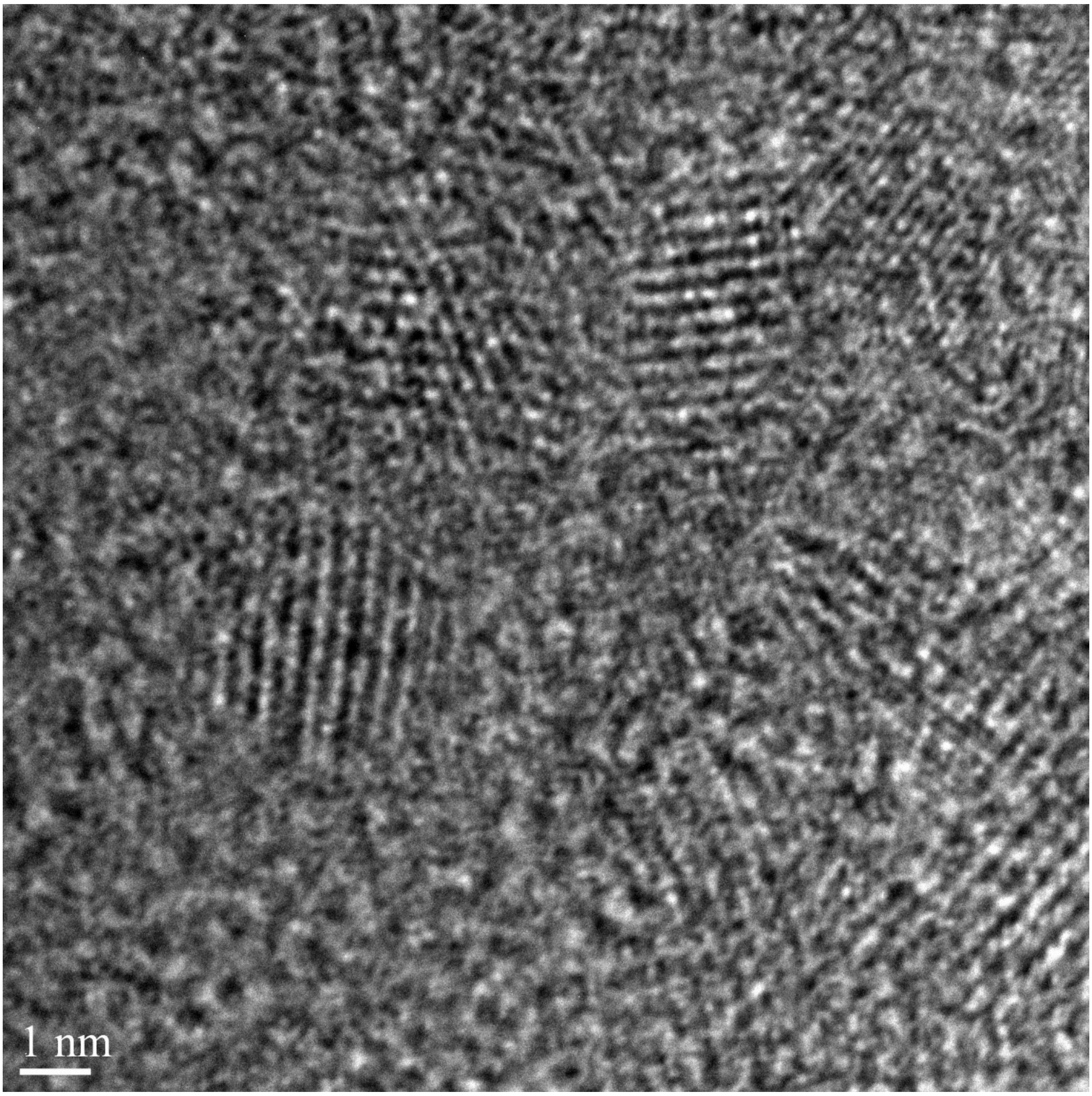

Fig. S4 HRTEM images of PAAMA-coated ultrasmall CeO_2 nanoparticles.

4. EDS spectra

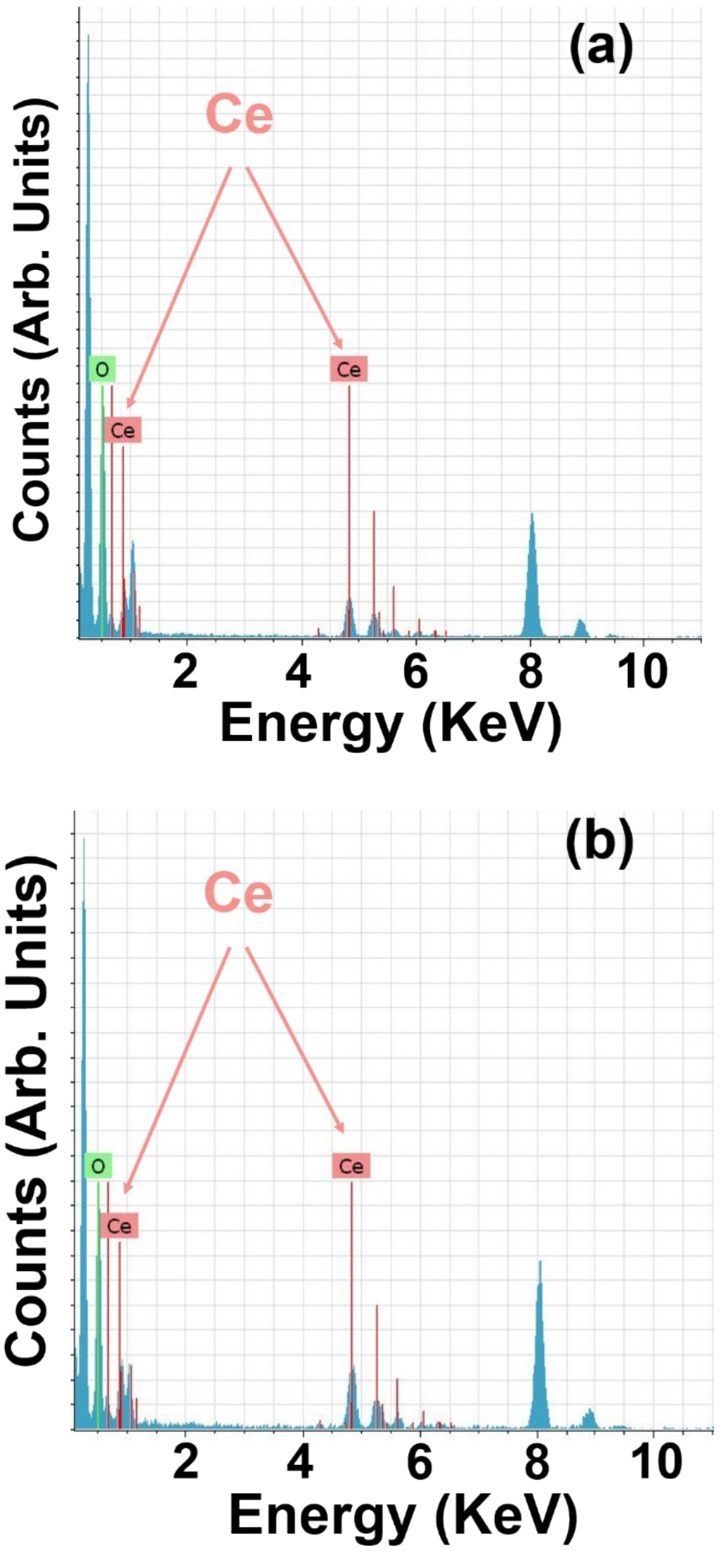

Fig. S5 EDS spectra of (a) PAA- and (b) PAAMA-coated ultrasmall CeO_2 nanoparticles.

5. Fourier transform-infrared (FT-IR) absorption frequencies

Table S1 Observed FT-IR absorption frequencies in cm^{-1}.

Vibration	PAA	PAAMA	PAA-CeO_2	PAAMA-CeO_2
C−H stretch	2943	2939	2929	2925
C=O stretch	1696	1694	1696	1694
COO^- antisymmetric stretch	-	-	1558 (138)[1]	1399 (295)[1]
COO^- symmetric stretch	-	-	1545 (151)[1]	1392 (302)[1]

[1] The values in parentheses indicate the red-shifts from C=O stretching vibrations.
